# Accurate crystal structure of ice VI from X-ray diffraction with Hirshfeld atom refinement

**DOI:** 10.1107/S2052252522006662

**Published:** 2022-07-16

**Authors:** Michal L. Chodkiewicz, Roman Gajda, Barbara Lavina, Sergey Tkachev, Vitali B. Prakapenka, Przemyslaw Dera, Krzysztof Wozniak

**Affiliations:** a Biological and Chemical Research Centre, Department of Chemistry, University of Warsaw, Żwirki i Wigury, Warszawa 02-089, Poland; b Advanced Photon Source, Argonne National Laboratory, 9700 South Cass Avenue, Lemont, IL 60439, USA; c Hawai’i Institute of Geophysics and Planetology, Université d’hawaï à mānoa, 1680 East-West Road, Honolulu, HI 96822, USA; University of St Andrews, United Kingdom

**Keywords:** deuterated ice, Hirshfeld atom refinement, quantum crystallography

## Abstract

Water is an essential chemical compound for living organisms, and twenty of its different crystal solid forms (ices) are known. Still, there are many fundamental problems with these structures such as establishing the correct positions and thermal motions of hydrogen atoms. The list of ice structures is not yet complete as DFT calculations have suggested the existence of additional and – to date – unknown phases. In many ice structures, neither neutron diffraction nor DFT calculations nor X-ray diffraction methods can easily solve the problem of hydrogen atom disorder or accurately determine their anisotropic displacement parameters (ADPs). Here, accurate crystal structures of H_2_O, D_2_O and mixed (50%H_2_O/50%D_2_O) ice VI obtained by Hirshfeld atom refinement (HAR) of high-pressure single-crystal synchrotron and laboratory X-ray diffraction data are presented. It was possible to obtain O—H/D bond lengths and ADPs for disordered hydrogen atoms which are in good agreement with the corresponding single-crystal neutron diffraction data. These results show that HAR combined with X-ray diffraction can compete with neutron diffraction in detailed studies of polymorphic forms of ice and crystals of other hydrogen-rich compounds. As neutron diffraction is relatively expensive, requires larger crystals which can be difficult to obtain and access to neutron facilities is restricted, cheaper and more accessible X-ray measurements combined with HAR can facilitate the verification of the existing ice polymorphs and the quest for new ones.

## Abstract 2

Accurate crystal structures of H_2_O, D_2_O and mixed (50%H_2_O/50%D_2_O) ice VI obtained by Hirshfeld atom refinement (HAR) of high-pressure single-crystal synchrotron and laboratory X-ray diffraction data are presented. It was possible to obtain O—H bond lengths and anisotropic displacement parameters for disordered hydrogen atoms which are in good agreement with the corresponding results of single-crystal neutron diffraction data. These results show that HAR combined with X-ray diffraction can compete with neutron diffraction in detailed studies of polymorphic forms of ice and crystals of other hydrogen-rich compounds.

## 1. Introduction

Water is a major component of the Earth’s hydro­sphere and living organisms. It forms different liquid forms such as aerosols and twenty different phases of solid ice. Water is essential for all known forms of life, it interacts with the most important biological substances such as proteins, DNA and polysaccharides, influencing protein folding, DNA base pairing and other crucial phenomena.

The first structural study on ice was probably Kepler’s essay ‘On the Six-Cornered Snowflake’ published in 1611 (Hoinkes, 1967 ▸). He used a round cannon ball arrangement metaphor to explain the creation of the different shapes of snowflakes, *i.e.* ice crystals (hexagonal ice I_h_ in modern terminology). The next major breakthrough was made possible by the studies of the Braggs and Max von Laue (Röntgen, 1898 ▸) of ice crystals with some of the earliest applications of X-ray diffraction (Rinne, 1917 ▸).

Currently, twenty different crystalline forms of solid ice are known (see the supporting information) as well as two amorphous solid forms (Chaplin, 2019 ▸). Among them, one can distinguish three metastable phases (ice IV, XII and XVII). Hydrogen atoms are ordered in eight other phases (ice II, VIII, IX, X, XI, XIII, XIV, XV) and there are nine phases in which disordered hydrogens are observed (ice I_h_, I_c_, I_sd_, III, V, VI, VII, XVI, XVIII). Ice X is not considered a disordered phase since the hydrogen atoms are located half-way between the oxygen atoms. Ice I_sd_ is a stacking disordered form of ice I (Malkin *et al.*, 2014 ▸). However, even more ice phases are believed to exist (Hansen, 2021 ▸; Jovanović *et al.*, 2020 ▸; Militzer & Wilson, 2010 ▸; Gets *et al.*, 2021 ▸; Prakapenka *et al.*, 2021 ▸; Zhu *et al.*, 2020 ▸; Grande *et al.*, 2022 ▸). Accurate determination of ice structures is performed mostly using neutron diffraction because hydrogen atoms diffract neutrons more strongly than X-rays. However, X-ray diffraction (XRD) is more readily available, uses smaller samples and requires shorter measurement times. In this paper we test the possibility of accurate determination of ice structures with XRD using a recently developed methodology, which greatly improves the accuracy of hydrogen atom structural parameters (Capelli *et al.*, 2014 ▸; Woińska *et al.*, 2016 ▸, 2021 ▸; Chodkiewicz *et al.*, 2020 ▸; Sanjuan-Szklarz *et al.*, 2020 ▸) and apply it to the study of ice VI.

The structure of ice VI was first described by Kamb (1965 ▸) and its existence on Earth in diamond inclusions was later confirmed by Kagi *et al.* (2000 ▸). This is the lowest high-pressure ice phase which exists at room temperature and contains disordered hydrogen atoms (Kuhs *et al.*, 1984 ▸). The structure of ice VI has been examined by neutron powder diffraction (Kuhs *et al.*, 1984 ▸; Salzmann *et al.*, 2016 ▸; Fortes *et al.*, 2012 ▸) and neutron single-crystal diffraction (Ahsbahs *et al.*, 1990 ▸; Kuhs *et al.*, 1989 ▸, 1996 ▸). More recently, X-ray single-crystal measurements of ice VI on regular diffractometers (Komatsu *et al.*, 2011 ▸) and near-infrared spectra of high-pressure ices (including ice VI) have been reported (Tonauer *et al.*, 2021 ▸). Inelastic neutron spectra of ice VI (also ice XV and XIX) have been also described (Rosu-Finsen *et al.*, 2020 ▸). For some high-pressure phases, a correspondence exists between a high-temperature disordered phase and an ordered lower-temperature phase, leading to attempts to predict the structure of the ordered analog of ice VI (Knight & Singer, 2005 ▸; Fan *et al.*, 2010 ▸; Kuo & Kuhs, 2006 ▸). Additionally, the oxygen topology was found to be the same in ice VI, XV (Salzmann *et al.*, 2009 ▸) and XIX (Gasser *et al.*, 2021*a* ▸, 2018 ▸; Yamane *et al.*, 2021 ▸; Salzmann *et al.*, 2021 ▸). While disordered phase VI, ordered phase XV and partially ordered phase XIX form a kind of family of ice structures where transitions from ordered to ordered, ordered to disordered, and disordered to ordered are possible (Gasser *et al.*, 2021*b* ▸). Structural distortions are claimed to be the main structural feature of ice XIX (Salzmann *et al.*, 2021 ▸).

Despite the fact that crystal structures of many ice phases have been known for a number of years, the exact positions of the hydrogens, as well as the hydrogen bonding parameters are still needed to accurately predict the possible phase transitions, This information has mostly been sought with density functional theory (DFT) (Fan *et al.*, 2010 ▸; Kuo & Kuhs, 2006 ▸; Brandenburg *et al.*, 2015 ▸; Kambara *et al.*, 2012 ▸; Xiao *et al.*, 2020 ▸). Research has focused on specific goals such as the creation of efficient algorithms to generate hydrogen-bond arrangements (Matsumoto *et al.*, 2021 ▸, 2018 ▸), and trying to predict the full phase diagram of water from first principles (Reinhardt & Cheng, 2021 ▸; Lu *et al.*, 2020 ▸). However, establishing the true positions and describing the thermal motion of disordered hydrogen atoms in ice structures remains a challenge. Among the 23 structures of solid ice deposited in the Inorganic Crystal Structure Database (Hellenbrandt, 2004 ▸), in 7 structures the positions of H/D atoms are not determined, and in 10 only isotropic displacement parameters for H/D atoms were refined. The displacement parameters of the atoms in ice are not only influenced by thermal effects. The orientational disorder may also cause uncertainty in the atom positions. This holds for the hydrogen atoms but also for the oxygen atoms. The displacements attributed to orientational disorder are described with spherical harmonics (Nelmes *et al.*, 1998 ▸).

A significant limitation of modern structural ice research is related to the fact that most polymorphic forms of ice can be accessed through changes in pressure. These pressures require the use of diamond anvil cells (DACs). Unfortunately, the restricted access to reciprocal space in DACs significantly reduces the amount of information (the number of available reflections) obtainable from these experiments. As a result, such experiments usually exhibit low data completeness.

**Figure 1:**
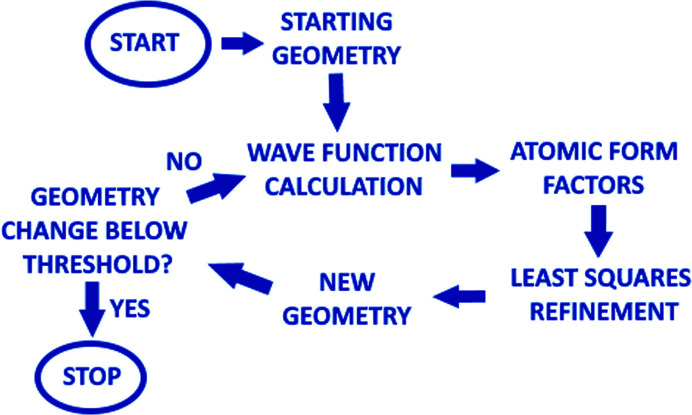
Scheme of the HAR procedure.

Although XRD is the most widely used diffraction technique in regular measurements, it has not been the first method of choice in ice phase investigations. XRD-determined covalent bond lengths to hydrogen atoms were typically 10–15% too short for organic molecules (Woińska *et al.*, 2016 ▸; Sanjuan-Szklarz *et al.*, 2020 ▸) and hydrogen displacement parameters were determined only in isotropic form. This was mainly due to the relatively low X-ray scattering power of hydrogen atoms and the limitations of the commonly used independent atom model (IAM) which assumes a spherical symmetry for the atomic electron densities. More advanced models incorporating aspherical atomic electron densities allowed for much more accurate and precise determinations of hydrogen atom positions and thermal parameters. One of the most promising methods is the Hirshfeld atom refinement (HAR) (Capelli *et al.*, 2014 ▸; Jayatilaka & Dittrich, 2008 ▸). It is based on quantum mechanical calculations of the electron density. Atomic form factors in this model are calculated from atomic contributions to the electron densities obtained by applying the Hirshfeld partition (Hirshfeld, 1977 ▸), but other partitions also can be used (Chodkiewicz *et al.*, 2020 ▸). The form factors are then further used in the least-squares refinement and improved structures are obtained. Next, the process continues as the calculated electron density provides further sets of atomic form factors to the point where convergence is achieved (see Fig. 1 ▸).

There are also other methods suitable for deriving structural parameters of hydrogen atoms. The transferable aspherical atom model (TAAM) utilizes the concept of transferability of atomic electron densities (Brock *et al.*, 1991 ▸) expressed in terms of the Hansen–Coppens multipole model. Libraries of extremely localized molecular orbitals (ELMOs) (Meyer & Genoni, 2018 ▸; Macetti & Genoni, 2019 ▸) apply this concept to molecular orbitals which, in combination with HAR methods, leads to the HAR–ELMO method (Malaspina *et al.*, 2019 ▸). ELMOs can be also used in the more advanced technique called HAR–QM/ELMO (Wieduwilt *et al.*, 2021 ▸) where ELMOs describe the crystal environment and the ‘central part’ of the system is treated quantum mechanically (as in HAR).

Both TAAM (Pichon-Pesme *et al.*, 1995 ▸; Domagała *et al.*, 2012 ▸; Dittrich *et al.*, 2004 ▸; Bąk *et al.*, 2011 ▸; Kumar *et al.*, 2019 ▸), HAR–ELMO and HAR–QM/ELMO can be used in crystallographic refinement to obtain accurate structural parameters for hydrogen atoms. Though HAR is typically slower than TAAM or HAR–ELMO, it does not rely on the concept of transferability and takes intermolecular interactions into account (which is not yet possible/implemented for TAAM and HAR–ELMO). This makes HAR slightly more accurate than the other two methods (at least in the case of small-molecule structures). Two versions of HAR allow for refinement of structures of disordered crystals (Kleemiss *et al.*, 2021 ▸; Chodkiewicz *et al.*, 2022 ▸). In this study, a locally developed *DiSCaMB* version of HAR was used for all HAR refinements (Chodkiewicz *et al.*, 2020 ▸). Approaches that are suitable for obtaining accurate positions and displacement parameters for hydrogen atoms utilizing XRD have not yet been used in studies of different ices with the exception of one experimental charge density study (vanBeek *et al.*, 1996 ▸). In this work we want to examine the usefulness of HAR for studies of high-pressure ice structures including ice VI, deuterated ice VI and mixed (50%H_2_O/50%D_2_O) ice VI.

### 1.1. Structure of ice VI

The structure of ice VI is formed from liquid water in the 0.6–2.2 GPa pressure range at 270 K (Dunaeva *et al.*, 2010 ▸). So far, many experimentors have determined the structure of ice VI under various pressures and temperatures. For example, its deuterated variant D_2_O was described by Kuhs *et al.* (1989 ▸) [*p* = 0.85 GPa, *T* = 296 (1) K, *a* = 6.21 Å, *c* = 5.73 Å] and also by Kuhs *et al.* (1984 ▸) (*p* = 1.1 GPa, *T* = 225 K, *a* = 6.1812 Å, *c* = 5.698 Å), and its hydrogenated variant by Kamb (1965 ▸) [ambient pressure, *T* = 98 K, *a* = 6.27 (1) Å, *c* = 5.79 (1) Å] and Komatsu *et al.* (2011 ▸) [*p* = 0.95 (5) GPa, *T* = 298 (1), *a* = 6.1990 (14) Å, *c* = 5.698 (3) Å].

**Figure 2:**
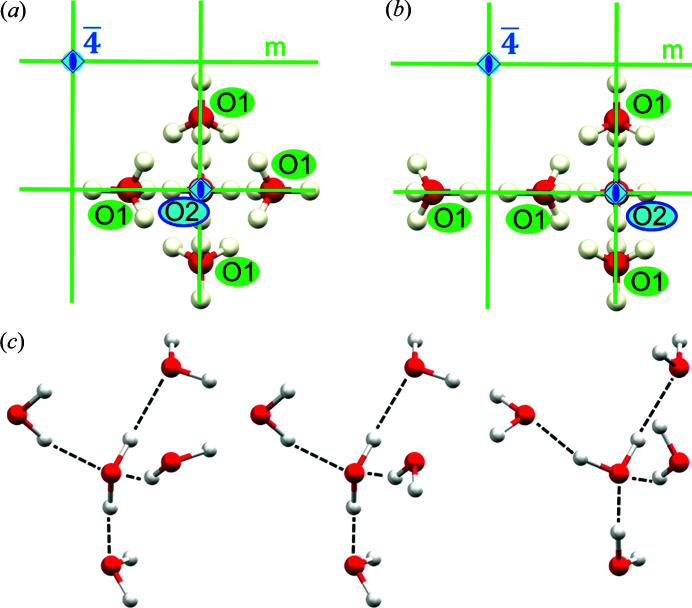
Selected symmetry elements in the structure of ice VI. Glide planes and inversion centers were removed for clarity: (*a*) O2 and its closest environment, (*b*) O1 and its closest environment, (*c*) examples of water clusters selected out of 259 configurations used in the HAR.

Ice VI forms tetragonal crystals with *P*4_2_/*nmc* (No. 137) space group symmetry. Water molecules connected by hydrogen bonds form two separate interpenetrating but non-interconnecting frameworks [see Fig. S2 of the supporting information]. There are two distinct types of water molecules and four distinct types of hydrogen bonds (Kuo & Kuhs, 2006 ▸). As depicted in Fig. 2 ▸, the oxygen atoms and most of the hydrogen atoms are placed at special positions.

**Figure 3:**
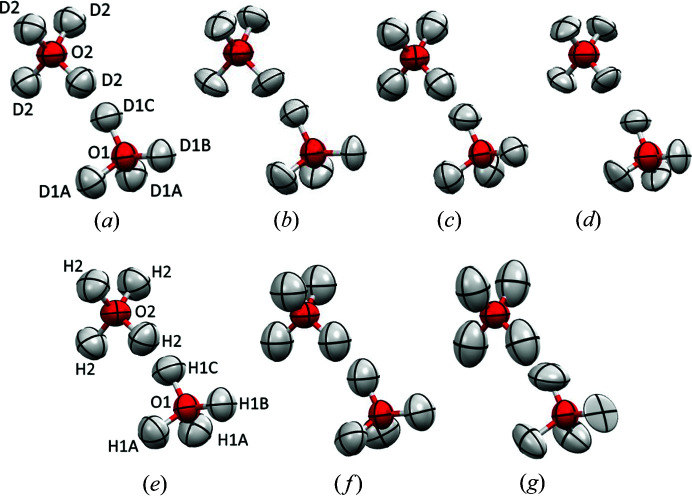
Disordered (*a*)–(*d*) D_2_O and (*e*)–(*g*) H_2_O molecules with their anisotropic thermal ellipsoids derived from (*a*) neutron diffraction, (*b*) HAR of synchrotron X-ray data, (*c*) HAR of laboratory X-ray source data, and (*d*) IAM refinement of synchrotron X-ray data, (*e*) neutron data, (*f*) HAR of laboratory source X-ray data, and (*g*) mixed ice (50%D_2_O/50%H_2_O) HAR of laboratory X-ray source data.

Each molecule of water forms hydrogen bonds to the four neighboring water molecules. In the average unit cell, each hydrogen atom has partial occupancy (0.5) and neighbors another hydrogen in close proximity. In the real structure, only one of the two atoms is present [*e.g.* either D1C or D2 in a given cluster, see Fig. 3(*a*) ▸] and only two of the four hydrogen sites surrounding each oxygen are occupied. These two conditions are known as ice rules or Bernal–Fowler rules (Ortiz-Ambriz *et al.*, 2019 ▸; Bernal & Fowler, 1933 ▸). There are many arrangements of hydrogen atoms which can satisfy these rules. For each of the oxygen atoms, it is possible to construct six configurations of the surrounding hydrogen atoms. In the case of O1, all of the configurations are equally probable (a consequence of the symmetry of the structure). For O2, which is surrounded by four symmetry equivalent hydrogen sites, there are two types of configurations differing in the H—O—H angle [see Fig. S2]. DFT calculations suggest that the probability of these configurations is close to equal at room temperature (Kuo & Kuhs, 2006 ▸) and we have used this assumption in the HAR.

### 1.2. HAR procedure

Since the structure of ice VI is disordered, the modeling of scattered intensities with HAR requires calculation of the electron density for multiple conformations of the system. There are four symmetry-independent arrangements of hydrogens around the O1 atom and two arrangements in the case of O2. The simplest approach (referred to hereafter as model 1) would require quantum mechanical calculations for the 4 + 2 configurations. However, when taking into account also the first-neighbor water molecules (model 2), the number of symmetry-independent configurations rapidly grows to 190 + 69 = 259. This approach assumes calculations of wavefunction for clusters built from five water molecules [see three selected examples of such clusters in Fig. 2(*c*) ▸]. In both models we also used point charges and dipoles to represent Coulomb interactions with the surrounding molecules. Details of the HAR calculations and determination of the water molecule configurations are included in the supporting information.

Atomic form factors are calculated using atomic electron densities of atoms of the water molecule averaged over all clusters:

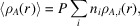

where ρ_*A*_(*r*) is the averaged electron density of atom *A*. The summation runs over all possible symmetry-independent configurations of the system, *P* is the configuration probability (assumed to be the same for all configurations), *n_i_* is the number of configurations which are symmetry equivalent with the *i*th configuration and ρ_*A*,*i*_(*r*) is the atomic electron density for atom *A* in this configuration. In the case of model 2 only the electron densities of the atoms of the central molecule are considered.

Model 2 is expected to be more accurate than model 1 since it treats a larger fragment of the structure quantum mechanically. We have tested both approaches and the difference is relatively small (the average difference in bond lengths is only 3 mÅ, the standard deviation for the bond lengths is about 15 mÅ). Data for model 1 are included in the supporting information. We have not attempted to perform calculations with larger clusters including second-neighbor water molecules.

## 2. Results

### 2.1. D_2_O ice VI

The heavy ice VI structures from HAR using data collected at both synchrotron and in-house sources have similar structural parameters to those obtained from neutron diffraction (Table 1 ▸). The average differences in bond lengths are 19 and 16 mÅ, respectively, with bond length uncertainties of about 16 mÅ for X-ray and 1.5 mÅ for neutron data. For comparison, we used the most recent structure of deuterated ice VI determined by Kuhs *et al.* (1989 ▸) using single-crystal neutron diffraction (Kuhs *et al.*, 1989 ▸). The O—D bond lengths resulting from HAR of the X-ray data obtained with a laboratory diffractometer (the D_2_O ice VI case) are in excellent agreement with the neutron reference values (within experimental uncertainties; see Table 1 ▸). The similarity between anisotropic displacement parameters (ADPs) measured with X-ray and neutron diffraction is also relatively high, especially for the in-house source measurements, yet some qualitative differences can be easily spotted (see Fig. 3 ▸). We have compared these (Table 1 ▸) using values from the similarity index S_12_ (see the supporting information for definitions of the descriptors) as introduced by Whitten & Spackman (2006 ▸), an average absolute difference and relative difference in ADP tensor components. It has been suggested that very good agreement for ADP values was achieved for S_12_ that is equal to or smaller than 1 with an in-house source measurement refined with HAR (Wanat *et al.*, 2021 ▸). Good agreement between X-ray and neutron structures should not be taken for granted as HAR is very sensitive to the quality of the X-ray data. A synchrotron measurement at a different source (DESY) gave a much larger average discrepancy in *X*—H bond length equal to 60 mÅ and significantly less reasonable ADP values (see the supporting information).

Since the refinement only provides information on the average structure of ice (*i.e.* the picture we observe is a combination of the various possible configurations of water molecules in the structure), the O—D distances and D—O—D angles do not correspond to the distances in the real, local structure of ice VI which, in each case, is one of the 259 clusters. However, single-crystal XRD gives an averaged structure of all 259 clusters. As a result, there are two quite different D—O—D angles involving the O2 atom: 95 and 115°.

Refinement with synchrotron data and the ‘classical model’, *i.e.* using spherical atomic electron densities (IAM), gave O—D distances that were significantly shorter (133 mÅ on average) than those from neutron the measurement. Interestingly, it was possible to refine the anisotropic displacement parameters for deuterium atoms and they were visually quite similar to those obtained from HAR (see Fig. 3 ▸).

We also want to add that, during the course of data refinement, we could also see some positional disorder for oxygen atoms; however, the quality of X-ray data seemed insufficient to resolve and refine this disorder.

### 2.2. H_2_O ice VI

The structure of normal H_2_O ice VI from HAR using data collected using an in-house source is less similar to the neutron structure – the difference in the bond lengths was 31 mÅ on average and the qualitative differences in ADP values were even easier to identify (Fig. 3 ▸). Yet it was in much better agreement than the structure from the IAM refinement (for both D_2_O and H_2_O; for the H_2_O data, refer to the supporting information).

### 2.3. Mixed H_2_O:D_2_O (1:1) ice VI

The bond lengths in the ice VI structure for the 1:1 mixture of normal and heavy water ice VI obtained from HAR using data collected with an in-house source are included in Table 1 ▸. There is no reference neutron data in this case, but this structure is likely to be less accurately determined than the H_2_O and D_2_O structures, as suggested by the large spread of O—H bond lengths (between 0.886 and 0.997 Å) and the shape of the ADPs [see Fig. 3 ▸(*g*)]. However, non-typical bond lengths and shapes of ADPs are not uncommon for disordered structures.

## 3. Conclusions

In summary, we have demonstrated that it is possible to successfully use XRD for accurate determination of the high-pressure, disordered structure of ice VI. This type of structural analysis is challenging, since X-rays are scattered from electrons and, therefore, they ‘see’ hydrogen atoms (with only one electron) less clearly than heavier atoms. The situation is even worse in the case of disordered structures with partial occupancies of hydrogen sites. Fortunately, in the case of ice VI the fractional occupancies of the hydrogen sites are known to be at least 0.5, whereas in other cases such as ice III or V occupancy deviates from 0.5 and must also be refined. An additional challenge is related to high-pressure measurements as they require the use of DACs. This restricts access to the reciprocal space and, as a consequence, diminishes the completeness and redundancy of the collected data. Yet with progress in experimental high-pressure techniques, an increase of data collection quality, and improved methods of experimental data analysis and interpretation, it is now possible to solve these kinds of problems with XRD. This certainly opens new possibilities for exploration of the phase diagram of water. Our results from high-pressure single-crystal synchrotron and in-house source X-ray data collection, combined with HAR, obtained for H_2_O ice VI and deuterated ice VI structures under pressure, resulted in bond lengths and ADP values which are in good agreement with the corresponding single-crystal neutron diffraction data. The average differences in O—H bond lengths are equal to 0.019 Å for synchrotron data for D_2_O, 0.016 Å for in-house source data for D_2_O and 0.031 Å for H_2_O ice VI. It was also possible to obtain hydrogen ADPs similar to those obtained from neutron diffraction. However, some qualitative differences were visible and for some datasets the ADPs from X-ray data were quite different than those from neutron diffraction. The results show that it is possible to obtain an accurate structure for high-pressure disordered ice from XRD combined with HAR refinement, but this cannot be taken for granted since it has a strong dependence on the quality of the collected data.

## 4. Methods

### 4.1. Preparation of single crystals

Single crystals of ice VI, deuterated ice VI and mixed (50%D_2_O/50%H_2_O) ice VI were obtained in a DAC (Fig. S7). The pressure chamber was filled with D_2_O. Pressure in the DAC chamber was gradually increased until the sample froze under isothermal conditions in the polycrystalline form. By increasing the temperature, all crystal grains except one melted. Then the temperature was slowly lowered until this single crystal grew under isochoric conditions to entirely fill the pressure chamber at room temperature.

The experimental details for all experimental data ice VI D_2_O conducted on in-house diffractometers as well as on synchrotron beamlines at the Advanced Photo Source (Argonne National Laboratory, USA) are presented in Table S1 of the supporting information.

### 4.2. In-house X-ray diffraction measurements

The pressure was determined with the ruby-fluorescence method (Piermarini *et al.*, 1975 ▸). The DAC with the ice VI sample was centered on a SuperNova diffractometer by the gasket-shadow method (Budzianowski, 2004 ▸). X-ray measurements were conducted at room temperature. The images were processed with *CrysAlis PRO* (Rigaku OD, 2014 ▸). The structures were solved and refined with *ShelXS* (Sheldrick, 2008 ▸) and *Olex2*, respectively, within the *Olex2* suite (Dolomanov *et al.*, 2009 ▸). For details of structural and data collection parameters, see the supporting information.

### 4.3. Synchrotron X-ray diffraction measurements

Data were collected in 15 batches, each characterized by a different chi position over the range 0–330°. In each batch, four runs with different exposure times (1, 10, 25 and 50 s) were recorded. Frames collected in each particular batch were processed together. Finally, data from all 15 batches were merged to form one *hkl* file. For details of structural and data collection parameters, see the supporting information.

### 4.4. Data analysis

For HAR, a locally modified version of *Olex2* (Dolomanov *et al.*, 2009 ▸) was used in the refinements incorporating a development version of *discamb2tsc* (Chodkiewicz *et al.*, 2020 ▸; Kumar *et al.*, 2019 ▸; Gildea *et al.*, 2011 ▸) based on the *DiSCaMB* library (Chodkiewicz *et al.*, 2018 ▸) which generates files with atomic form factors in tsc format (Kleemiss *et al.*, 2021 ▸; Midgley *et al.*, 2019 ▸). Such files are then imported into *Olex2* and used in the refinement. Details of the implementation are given by Chodkiewicz *et al.* (2022 ▸). A density functional method with a B3LYP functional and cc-pVTZ basis set was used for calculation of the electron densities. Quantum mechanical calculations were performed with *ORCA* (Neese *et al.*, 2020 ▸).

### 4.5. Data availability

The data supporting the findings of this study are available from the corresponding authors upon reasonable request. The crystal structures reported in this study have been deposited via the joint CCDC/FIZ Karlsruhe deposition service with the CCDC numbers 2160500–2160510. A computer code used in this study is available upon request from MC.

## 5. Related literature

The following references are cited in the supporting information: Komatsu *et al.* (2016 ▸); Rosu-Finsen & Salzmann (2019 ▸); Salzmann (2019 ▸); Shephard & Salzmann (2015 ▸); Thoeny *et al.* (2019 ▸); Whale *et al.* (2013 ▸).

## Supplementary Material

Crystal structure: contains datablock(s) D2O_APS_HAR_mod1, D2O_APS_HAR, D2O_APS_IAM, D2O_DESY_HAR, D2O_DESY_IAM, D2O_home_HAR, D2O_home_IAM, H2O_D2O_home_HAR, H2O_D2O_home_IAM, H2O_home_HAR, H2O_home_IAM. DOI: 10.1107/S2052252522006662/lt5051sup1.cif

Supporting figures and tables. DOI: 10.1107/S2052252522006662/lt5051sup2.pdf

CCDC references: 2160500, 2160502, 2160503, 2160504, 2160505, 2160506, 2160507, 2160508, 2160510, 2189429, 2189430

## Figures and Tables

**Table 1:** Comparison of bond lengths and hydrogen ADPs in ice VI For ADP values see the supporting information. 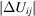 is the average absolute difference between ADP components, 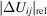 is an analogous value, taking into account the relative difference of each ADP but is divided by the average value and S_12_ is the average similarity index for the hyrogen ADPs.

	D_2_O ice VI				H_2_O		H_2_O:D_2_O (1:1)
Radiation	Neutron	X-ray			Neutron	X-ray	X-ray
Source		Synchrotron	Laboratory	Synchrotron		Laboratory	In-house
Model		HAR	HAR	IAM		HAR	HAR
Bond lengths							
O2—H2 (Å)	0.932 (1)	0.951 (15)	0.913 (18)	0.806 (13)	0.932 (4)	0.899 (20)	0.927 (50)
O1—H1A (Å)	0.942 (1)	0.947 (12)	0.930 (13)	0.797 (9)	0.942 (3)	0.919 (15)	0.997 (30)
O1—H1B	0.945 (1)	0.988 (15)	0.932 (20)	0.819 (13)	0.947 (3)	0.928 (20)	0.967 (40)
O1—H1C	0.932 (2)	0.954 (15)	0.917 (17)	0.810 (13)	0.938 (4)	0.891 (20)	0.886 (50)

Average difference in bond lengths between the neutron and X-ray structures (Å)							
		0.019	0.016	0.133		0.031	

Average difference in hydrogen ADPs in the neutron and X-ray structures							
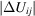		0.0056	0.0040	0.0062		0.0075	
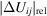		0.23	0.17	0.23		0.28	
S_12_		1.48	0.61	2.65		1.52	
