# Supplementary material for: Accurate crystal structure of ice VI from X-ray diffraction with Hirshfeld atom refinement
Source: IUCrJ. 2022 Jul 16;9(Pt 5):573–9. doi: 10.1107/S2052252522006662 (PMC9438488; doi:10.1107/S2052252522006662)
Supplement: Supplementary file 2 [file m-09-00573-sup2.pdf]

IUCrJ

Volume 9 (2022)

Supporting information for article:

Accurate crystal structure of ice VI from X-ray diffraction with Hirshfeld atom refinement

Michal L. Chodkiewicz, Roman Gajda, Barbara Lavina, Sergey Tkachev, Vitali B. Prakapenka, Przemyslaw Dera and Krzysztof Wozniak

Accurate crystal structure of ice VI from X-ray diffraction with Hirshfeld Atom Refinement

Michał L. Chodkiewicz[1*], Roman Gajda[1], Barbara Lavina[2], Sergey Tkachev[2], Vitali B. Prakapenka[3], Przemysław Dera[3], Krzysztof Woźniak[1*]

[1]Biological and Chemical Research Centre, Department of Chemistry, University of Warsaw, Żwirki i Wigury 101, Warszawa, 02-089, Poland.

[2]Advanced Photon Source, Argonne National Laboratory, 9700 South Cass Avenue, Building 434, Room A007 Lemont, IL 60439, USA.

[3]Hawai‘i Institute of Geophysics and Planetology, University of Hawai‘i at Mānoa, 1680 East-West Road, POST Building, Office 602, Honolulu, HI 96822, USA

Mail: ml.chodkiewicz@uw.edu.pl, kwozniak@chem.uw.edu.pl

Contents

1. **Phase transitions of ice VI**
2. **Data collection and ice VI crystal structure parameters**
3. **ADP values**
   (a) As refined
   (b) Diagonal tensor representation
4. **Definitions of ADP similarity descriptors**
5. **Visualisation of ice VI structure**
6. **Generation of the configurations and use of point multipoles**
7. **DESY structure of ice VI**
8. **IAM structure of H_2O ice VI**
9. **APS structure of ice VI for HAR with model 1**
10. **References**

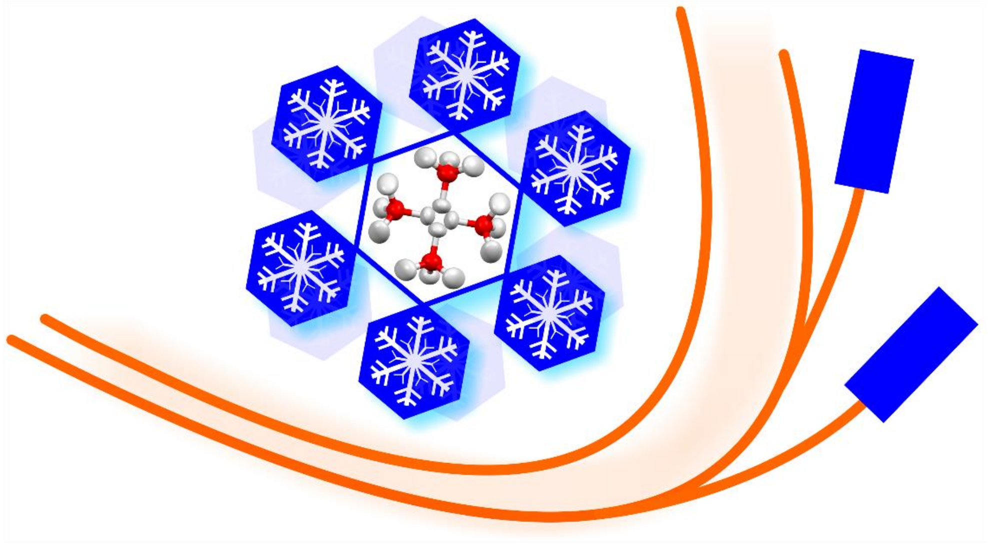

1. Phase transitions of ice VI

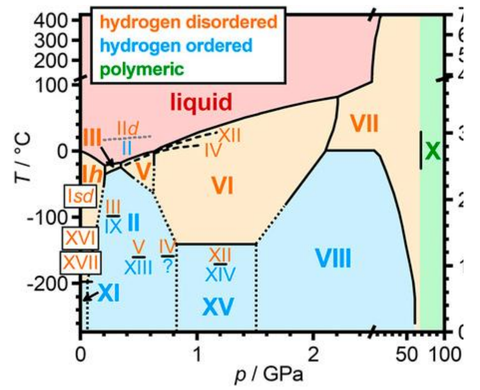

Figure 1S. Phase diagram of ice including ice VI; the H-disordered, and H-ordered phases of ice are in orange and blue respectively. Stable phases are highlighted by large bold Roman numerals, whereas metastable states are indicated by a smaller font size.

Hydrogen disorder is frozen-in upon cooling pure ice VI.(Kuhs *et al.*, 1984) The use of hydrochloric acid (HCl) as a dopant led to the discovery of the antiferroelectric hydrogen-ordered ice XV.(Militzer & Wilson, 2010) Upon cooling HCl-doped ice VI at pressures above ~1 GPa, the hydrogen-ordering phase transition from ice VI to ice XV is suppressed.(Salzmann *et al.*, 2009, 2016; Whale *et al.*, 2013; Shephard & Salzmann, 2015; Komatsu *et al.*, 2016) These doped samples are referred to as deep-glassy ice VI.(Rosu-Finsen & Salzmann, 2019) Upon heating at ambient pressure, deep-glassy ice VI exhibits transient ordering as the metastable ice VI first undergoes irreversible hydrogen ordering to ice XV and next reversible hydrogen disordering to ice VI.(Salzmann *et al.*, 2016; Whale *et al.*, 2013; Shephard & Salzmann, 2015) It was shown recently, that HCl-doped ice VI samples cooled at pressures above 1.4 GPa display an endothermic feature before the exothermic transient ordering upon heating at ambient pressure and certain heating rates,(Rosu-Finsen & Salzmann, 2019; Gasser *et al.*, 2018) which seems to arise from a new hydrogen-ordered phase of ice with different hydrogen ordering-type and more stable than ice XV.(Gasser *et al.*, 2018; Thoeny *et al.*, 2019) However, it has also been pointed out that the new endotherm is consistent with a kinetic overshoot effect associated with the glass transition of deep glassy ice VI.(Rosu-Finsen & Salzmann, 2019) Upon increasing the pressure, more relaxed states are obtained. A Raman spectroscopic study,(Thoeny *et al.*, 2019) combined inelastic neutron spectroscopy and neutron diffraction investigations of an HCl-doped ice VI slow-cooled at 1.7 GPa,(Rosu-Finsen *et al.*, 2020) showed that the sample was structurally very similar to standard pure ice VI. It was speculated that local and orientationally uncorrelated hydrogen ordering may be occurring as the pressure is increased. This could lead to increased levels of local distortions that could eventually even reduce the space group symmetry of ice VI.(Rosu-Finsen *et al.*, 2020) Structural confirmation of such subtle phase transition effects requires far better methods of refinement than spherical independent atom model refinements. Phase diagram of ice presented in Figure 1s was adopted from Salzmann (2019).(Salzmann, 2019)

2. Data collection details and ice VI crystal structure parameters

Table 1Sa. Experimental details of X-ray data collection and structure refinement corresponding with experiments conducted on laboratory diffractometers.

**Crystal data**			
Chemical formula	H_2O	D_2O	H_2O/D_2O
Crystal system, space group	Tetragonal, $P4_2/nmc$	Tetragonal, $P4_2/nmc$	Tetragonal, $P4_2/nmc$
Pressure (GPa)	1.5(1)	1.2(1)	1.5(1)
a, *c* (Å)	6.1665 (2), 5.6756 (4)	6.1918 (4), 5.7067 (4)	6.1737 (5), 5.6843 (3)
V (Å^3)	215.82 (2)	218.79 (3)	216.66 (3)
Z	10	10	10
F(000)	100	100	100
D_x (Mg m^{-3})	1.386	1.367	1.381
Radiation type	Ag $K\alpha$, λ = 0.56087 Å	Ag $K\alpha$, λ = 0.56087 Å	Ag $K\alpha$, λ = 0.56087 Å
No. of reflections for cell measurement	1521	1022	1312
θ range (°) for cell measurement	3.7–22.9	3.7–23.6	3.7–23.7
completeness	95% to 2θ=53.8°	89% to 2θ=53.5°	88% to 2θ=53.5°
μ (mm^{-1})	0.09	0.09	0.09
**Data collection**			
Diffractometer	SuperNova, Single source at offset/far, Eos	SuperNova, Single source at offset/far, Eos	SuperNova, Single source at offset/far, Eos
Radiation source	micro-focus sealed X-ray tube, SuperNova (Ag) X-ray Source	micro-focus sealed X-ray tube, SuperNova (Ag) X-ray Source	micro-focus sealed X-ray tube, SuperNova (Ag) X-ray Source
Monochromator	Mirror	Mirror	Mirror
Detector resolution (pixels mm^{-1})	16.0128	16.0128	16.0128
Scan method	ω scans	ω scans	ω scans
Absorption correction	Multi-scan	Multi-scan	–
T_{min}, T_{max}	0.685, 1.000	0.809, 1.000	-
No. of measured, independent, observed [$I > 2\sigma(I)$] reflections	4280, 259, 198	2579, 240, 182	3861, 238, 175
R_{int}	0.035	0.036	0.057
θ values (°)	θ_{max} = 26.9, θ_{min} = 3.7	θ_{max} = 26.7, θ_{min} = 3.7	θ_{max} = 26.8, θ_{min} = 3.7
$(\sin \theta/\lambda)_{max}$ (Å^{-1})	0.806	0.800	0.802
Range of *h*, *k*, *l*	$h = -9\rightarrow9$, $k = -8\rightarrow8$, $l = -8\rightarrow8$	$h = -9\rightarrow8$, $k = -9\rightarrow9$, $l = -9\rightarrow9$	$h = -9\rightarrow9$, $k = -8\rightarrow8$, $l = -9\rightarrow9$
Refinement			
Refinement on	F^2	F^2	F^2
$R[F^2 > 2\sigma(F^2)]$, $wR(F^2)$, S	0.017, 0.029, 1.04	0.016, 0.027, 0.94	0.031, 0.075, 1.16

No. of reflections	259	240	238
No. of parameters	22	22	22
No. of restraints	0	0	0
Weighting scheme	$w = 1/[\sigma^2(F_o^2) + (0.010P)^2 + 0.0017P]$ where $P = (F_o^2 + 2F_c^2)/3$	$w = 1/[\sigma^2(F_o^2) + (0.010P)^2 + 0.0009P]$ where $P = (F_o^2 + 2F_c^2)/3$	$w = 1/[\sigma^2(F_o^2) + (0.0262P)^2 + 0.0138P]$ where $P = (F_o^2 + 2F_c^2)/3$
$(\Delta/\sigma)_{max}$	0.0003	0.0001	< 0.001
$\Delta\rangle_{max}$, $\Delta\rangle_{min}$ (e Å^{-3})	+0.18, -0.16	+0.16, -0.14	+0.27, -0.28

Table 1Sb. Experimental details of X-ray data collection and structure refinement corresponding with experiments conducted on synchrotron facility.

**Crystal data**		
Facility	APS	DESY
Chemical formula	D_2O	D_2O
Crystal system, space group	Tetragonal, $P4_2/nmc$	Tetragonal, $P4_2/nmc$
Pressure (GPa)	1.1(1)	0.8(1)
a, *c* (Å)	6.1732 (3), 5.6881 (5)	6.1711 (3), 5.6747 (5)
V (Å^3)	216.76 (3)	216.11 (3)
Z	10	10
F(000)	100	100
D_x (Mg m^{-3})	1.380	1.384
Radiation type	synchrotron, λ = 0.27552 Å	Synchrotron, λ = 0.50014 Å
No. of reflections for cell measurement	922	594
θ range (°) for cell measurement	1.8–16.1	2.3–23.2
completeness	66% to 2θ=40.0°	76% to 2θ=45.9°
μ (mm^{-1})	0.03	0.07
**Data collection**		
Diffractometer	Esperanto-*CrysAlis PRO*-abstract goniometer imported esperanto images	Esperanto-*CrysAlis PRO*-abstract goniometer imported esperanto images
Radiation source	synchrotron	synchrotron
Monochromator	synchrotron	synchrotron
Detector resolution (pixels mm^{-1})		5.8140
Scan method	ϕ scans	ϕ scans
Absorption correction		Multi-scan *CrysAlis PRO* 1.171.40.67a (Rigaku Oxford Diffraction, 2019) Empirical absorption correction using spherical harmonics, implemented in SCALE3 ABSPACK scaling

		algorithm.
T_{min}, T_{max}		0.613, 1.000
No. of measured, independent, observed [$I > 2\sigma(I)$] reflections	629, 622, 364	1022, 191, 178
R_{int}	-	0.023
θ values (°)	θ_{max} = 20.0, θ_{min} = 1.8	θ_{max} = 23.0, θ_{min} = 3.3
$(\sin \theta/\lambda)_{max}$ (Å^{-1})	1.240	0.780
Range of *h*, *k*, *l*	$h = 1\rightarrow14$, $k = 0\rightarrow10$, $l = 0\rightarrow12$	$h = -7\rightarrow7$, $k = -7\rightarrow7$, $l = -7\rightarrow7$
Refinement		
Refinement on	F^2	F^2
$R[F^2 > 2\sigma(F^2)]$, $wR(F^2)$, S	0.031, 0.079, 1.40	0.078, 0.149, 0.94
No. of reflections	622	191
No. of parameters	36	22
No. of restraints	0	7
Weighting scheme	$w = 1/[\sigma^2(F_o^2) + (0.0217P)^2]$ where $P = (F_o^2 + 2F_c^2)/3$	$w = 1/[\sigma^2(F_o^2) + (0.1575P)^2]$ where $P = (F_o^2 + 2F_c^2)/3$
$(\Delta/\sigma)_{max}$	0.0003	0.002
$\Delta\rangle_{max}$, $\Delta\rangle_{min}$ (e Å^{-3})	0.30, -0.26	0.82, -0.53

3. ADP values

(a) As refined

Table 2S. ADPs as refined, HAR refinements with model 2 (unless stated otherwise)

	U_{11}	U_{22}	U_{33}	U_{12}	U_{13}	U_{23}
**D_2O/HAR (model 1)/synchrotron(APS)**						
**O1**	0.03106(14)	0.04247(16)	0.04129(19)	0.00000	0.00084(8)	0.000000
**O2**	0.04362(19)	0.04362(19)	0.0329(2)	-0.000000	-0.000000	-0.000000
**D1A**	0.047(7)	0.052(8)	0.049(6)	-0.002(6)	0.006(6)	0.025(6)
**D1B**	0.023(6)	0.036(8)	0.052(12)	0.000000	-0.003(4)	0.000000
**D2**	0.059(10)	0.041(10)	0.031(9)	0.000000	0.022(8)	0.000000
**D1C**	0.034(8)	0.044(9)	0.040(11)	0.000000	-0.001(6)	0.000000
**D_2O/HAR/synchrotron(APS)**						
**O1**	0.03078(13)	0.04247(15)	0.04117(18)	0.00000	0.00086(8)	0.00000
**O2**	0.04365(18)	0.04365(18)	0.0325(2)	0.00000	0.00000	0.00000
**D1A**	0.051(7)	0.053(7)	0.055(6)	-0.004(5)	0.006(6)	0.025(6)
**D1B**	0.026(5)	0.041(8)	0.057(11)	0.00000	-0.001(5)	0.00000
**D2**	0.058(10)	0.046(10)	0.037(10)	0.00000	0.019(7)	0.00000
**D1C**	0.040(9)	0.050(9)	0.037(10)	0.00000	-0.003(6)	0.00000
**D_2O/HAR/home**						
**O1**	0.0304(3)	0.0426(3)	0.0406(3)	0.00000	0.00111(18)	0.00000
**O2**	0.0438(4)	0.0438(4)	0.0320(4)	0.00000	0.00000	0.00000
**D1A**	0.044(7)	0.053(7)	0.057(7)	-0.003(5)	0.001(5)	0.005(6)
**D1B**	0.040(12)	0.054(11)	0.046(7)	0.00000	0.004(6)	0.00000
**D2**	0.054(13)	0.058(11)	0.054(10)	0.00000	-0.006(7)	0.00000
**D1C**	0.052(10)	0.059(11)	0.036(8)	0.00000	0.004(8)	0.00000
**D_2O/IAM/synchrotron(APS)**						
**O1**	0.03114(13)	0.04285(16)	0.04155(19)	0.00000	0.00085(8)	0.00000
**O2**	0.04401(19)	0.04401(19)	0.0328(2)	-0.000000	0.00000	0.00000
**D1A**	0.046(5)	0.035(5)	0.045(5)	0.001(4)	0.007(4)	0.022(5)
**D1B**	0.020(5)	0.041(7)	0.053(9)	-0.000000	0.002(4)	0.00000
**D2**	0.037(6)	0.049(8)	0.030(7)	-0.000000	0.015(5)	0.00000
**D1C**	0.040(7)	0.049(8)	0.025(9)	-0.000000	-0.001(5)	0.00000
**D_2O/HAR/synchrotron(DESY)**						
**O1**	0.0312(4)	0.0445(5)	0.0485(5)	-0.000000	0.0011(3)	0.00000
**O2**	0.0448(6)	0.0448(6)	0.0388(7)	-0.000000	0.00000	0.00000
**D1A**	0.0540	0.0540	0.0540	0.000000	0.00000	0.00000
**D1B**	0.13(4)	0.021(16)	0.11(3)	-0.000000	0.05(3)	0.00000
**D2**	0.06(2)	0.08(3)	0.11(4)	-0.000000	0.02(2)	0.00000
**D1C**	0.07(2)	0.09(3)	0.04(2)	-0.000000	0.004(19)	0.00000
**H_2O/HAR/home**						
**O1**	0.0291(3)	0.0403(3)	0.0397(3)	0.000000	0.0009(2)	0.00000
**O2**	0.0416(4)	0.0416(4)	0.0318(5)	0.000000	0.000000	0.00000
**D1A**	0.067(8)	0.045(7)	0.057(9)	-0.013(6)	-0.009(7)	0.000(6)
**D1B**	0.041(12)	0.080(15)	0.066(12)	0.000000	0.006(8)	0.00000
**D2**	0.046(13)	0.071(13)	0.075(18)	0.000000	-0.001(9)	0.00000
**D1C**	0.044(12)	0.068(14)	0.069(16)	0.000000	0.000(12)	0.00000
**H_2O:D_2O/HAR/home**						
**O1**	0.0307(7)	0.0431(6)	0.0399(6)	-0.000000	0.0007(4)	0.000000

**O2**	0.0437(8)	0.0437(8)	0.0334(8)	-0.000000	0.00000	0.000000
**D1A**	0.08(2)	0.034(17)	0.060(17)	-0.008(13)	0.027(17)	0.000(15)
**D1B**	0.01(2)	0.13(5)	0.07(3)	-0.000000	0.019(16)	0.000000
**D2**	0.06(4)	0.06(3)	0.10(5)	-0.000000	-0.01(2)	0.000000
**D1C**	0.07(4)	0.10(4)	0.04(2)	-0.000000	0.01(2)	0.000000

(b) Diagonal tensor representation

Table 3S. ADP values in the diagonal tensor representation, HAR refinements with model 2 (unless stated otherwise)

	U^D_{11}	U^D_{22}	U^D_{33}	$Ueq=1/3(U^D_{11}+ U^D_{22}+ U^D_{33})$
**D_2O/HAR (model 1)/synchrotron(APS)**				
**O1**	0.0425	0.0414	0.0310	0.0383
**O2**	0.0436	0.0436	0.0329	0.0400
**D1A**	0.0761	0.0476	0.0244	0.0494
**D1B**	0.0521	0.0357	0.0229	0.0369
**D2**	0.0713	0.0408	0.0195	0.0439
**D1C**	0.0440	0.0399	0.0336	0.0392
**D_2O/HAR/synchrotron(APS)**				
**O1**	0.0425	0.0412	0.0307	0.0381
**O2**	0.0436	0.0436	0.0325	0.0399
**D1A**	0.0797	0.0528	0.0270	0.0532
**D1B**	0.0571	0.0408	0.0261	0.0413
**D2**	0.0696	0.0461	0.0260	0.0472
**D1C**	0.0503	0.0421	0.0352	0.0425
**D_2O/HAR/home**				
**O1**	0.0426	0.0407	0.0303	0.0379
**O2**	0.0438	0.0438	0.0320	0.0399
**D1A**	0.0609	0.0512	0.0430	0.0517
**D1B**	0.0538	0.0478	0.0380	0.0465
**D2**	0.0602	0.0580	0.0482	0.0555
**D1C**	0.0588	0.0528	0.0355	0.0490
**D_2O/IAM/synchrotron(APS)**				
**O1**	0.0428	0.0416	0.0311	0.0385
**O2**	0.0440	0.0440	0.0328	0.0403
**D1A**	0.0645	0.0441	0.0172	0.0419
**D1B**	0.0532	0.0411	0.0199	0.0381
**D2**	0.0489	0.0486	0.0176	0.0384
**D1C**	0.0495	0.0398	0.0249	0.0381
**D_2O/HAR/synchrotron(DESY)**				
**O1**	0.0486	0.0445	0.0312	0.0414
**O2**	0.0448	0.0448	0.0388	0.0428
**D1A**	0.0540	0.0540	0.0540	
**D1B**	0.1732	0.0701	0.0212	0.0882
**D2**	0.1191	0.0826	0.0512	0.0843
**D1C**	0.0883	0.0723	0.0418	0.0675
**H_2O/HAR/home**				
**O1**	0.0403	0.0398	0.0290	0.0364

**O2**	0.0416	0.0416	0.0318	0.0383
**H1A**	0.0764	0.0540	0.0379	0.0561
**H1B**	0.0797	0.0669	0.0394	0.0620
**H2**	0.0751	0.0706	0.0458	0.0638
**H1C**	0.0687	0.0677	0.0443	0.0602
**H_2O:D_2O/HAR/home**				
**O1**	0.0432	0.0399	0.0307	0.0379
**O2**	0.0437	0.0437	0.0334	0.0403
**D1A/H1A**	0.0970	0.0427	0.0302	0.0566
**D1B/H1B**	0.1335	0.0733	0.0017	0.0695
**D2/H2**	0.1031	0.0594	0.0556	0.0727
**D1C/H1C**	0.0973	0.0780	0.0338	0.0697

4. Definitions of ADP similarity descriptors

Similarity Index S_{12}

A convenient method of comparison of ADP values is the similarity index[13]. This indicator was introduced to characterize the degree of agreement between ADP values obtained using approaches 1 and 2. It can be defined in terms of the overlap between the two probability density functions in the direct space. It is convenient to transform the ADP tensor U to a Cartesian system, and for two compared tensors U_1 and U_2 the equation has the form:(Whitten & Spackman, 2006)

$$R_{12} = \int [p_1(x)p_2(x)]^{\frac{1}{2}} d^3x = \frac{2^{\frac{3}{2}}(detU_1^{-1}U_2^{-1})^{1/4}}{[\det(U_1^{-1}+U_2^{-1})]^{1/2}}$$

The probability density functions are normalized, so as a result for $U_1 = U_2$, $R_{12} = 1.0$. To underline the subtle differences and present them as a percentage difference between two compared ADP values we used a similarity index in the form:

$$S_{12} = 100(1 - R_{12})$$

Average absolute difference in ADPs tensor components $\langle|\Delta U_{ij}|\rangle$

The descriptor for an individual atom is given as:

$$|\Delta U_{ij}| = \frac{1}{6}\sum_{i\le j} |U_{ij}^{X-ray} - U_{ij}^{n}|$$

where U_{ij}^{X-ray} corresponds to X-ray and U_{ij}^{n} to neutron diffraction structure. We report the value averaged over all (four) H-atoms in the asymmetric unit ($\langle|\Delta U_{ij}|\rangle$).

Relative average absolute difference in ADPs tensor components $\langle|\Delta U_{ij}|_{rel}\rangle$

The descriptor for an individual atom is given as:

$$\left|\Delta U_{ij}\right|_{rel} = \frac{\sum_{i \le j} \left|U_{ij}^{X-ray} - U_{ij}^{n}\right|}{\sum_{i \le j} \left|U_{ij}^{n}\right|}$$

We report the value averaged over all (four) H-atoms in the asymmetric unit ($\langle\left|\Delta U_{ij}\right|_{rel}\rangle$).

5. Visualization of ice VI structure

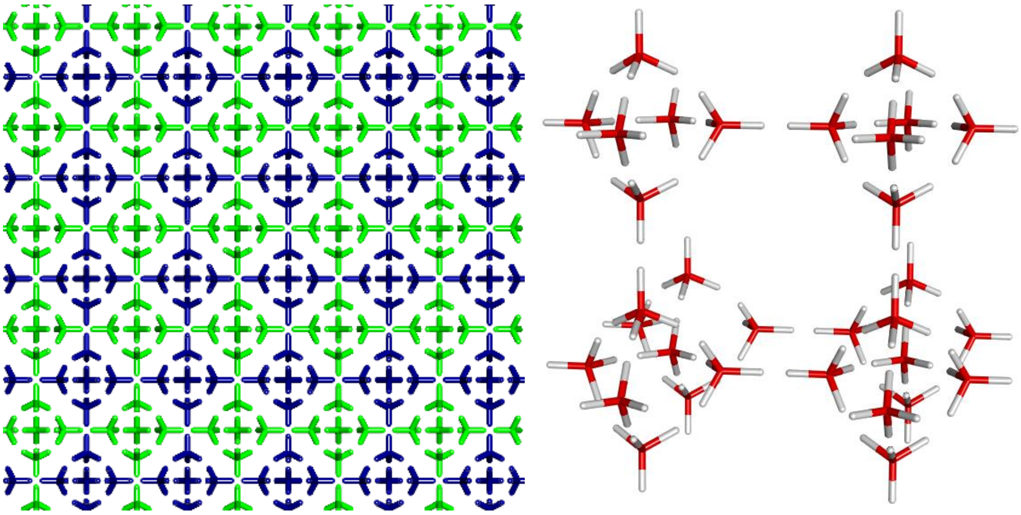

(a) (b)

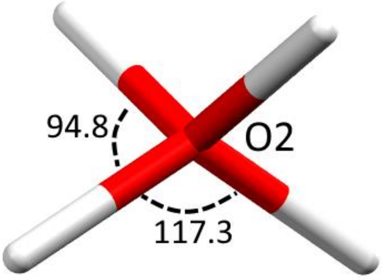

(c)

Figure 2S. Ice structure (a) two interpenetrating hydrogen bond networks in ice VI marked with different colors. The view is along the direction [001], (b) fragment of one of the networks, (c) two non-equivalent water configurations associated with O2 marked with the corresponding angles. The picture shows HAR derived structure of heavy ice VI (synchrotron data).

(6) Generation of the configurations and use of point multipoles

A commonly used representation of a water clusters as directed graph has been used (see Fig. 3S) for generation of all possible clusters of water (for fixed choice of participating oxygen atoms).

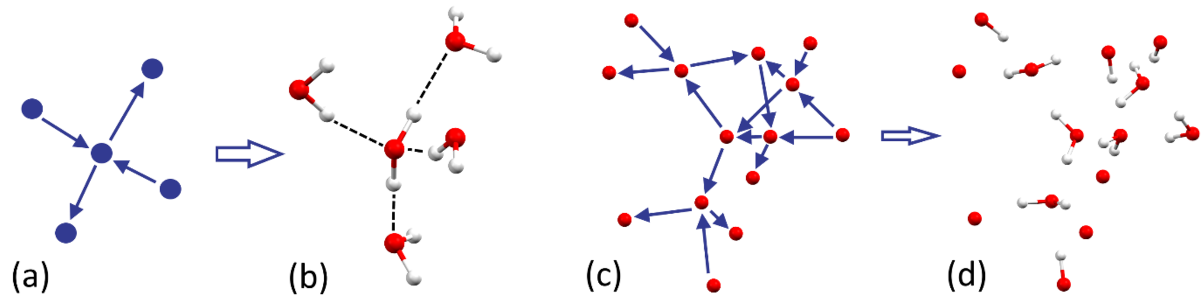

Figure 3S. Examples of mapping graphs and corresponding water clusters (see text).

Each hydrogen bond involves a hydrogen atom covalently bonded to one of two participating oxygen atoms. Due to disorder, it is possible that the hydrogen is bound to any of the two atoms. The two possibilities are represented with two possible directions of the edge in the graph. We can also represent the bond/edge 'direction' with two numbers ('bytes') - 0 and 1 and represent N such bonds as a N digit binary number. A brute force algorithm was used to generate all of the possible clusters (represented with numbers from 0 to (2^N-1)) and those fulfilling the ice rules were kept. Next the configurations were grouped into sets of symmetry related ones and only one in such a set was used for wave function calculations. The mapping from a graph (see e.g. Fig. 3S (a)) to a molecule Fig. 3S (b) does not define which of the outermost hydrogen atoms are present (they have been added arbitrarily in Fig. 3S (b)). Therefore, we have used a graph involving also the second neighbour molecules – see e.g. Fig. 3S (c) and the corresponding system containing only the hydrogens defined by the graph (Fig. 3S (d)).

HAR in this work involves quantum mechanical calculations with point charges and dipoles representing the electrostatic field of the surrounding molecules. In the calculations for clusters containing first neighbor water molecules (*i.e.* central water molecule + 4 neighboring molecules) treated quantum-mechanically, the atoms of the external (2nd) shell of water molecules were used as placeholders for placing the point charges and dipoles. The most external hydrogen atoms in this shell (the H-atoms missing in Fig 3S (d)) have partial occupation matching hydrogen bonding situation of given water molecule. The location of the external hydrogen atoms is not defined by the graph, yet it can be determined how many of them should be attached to a given oxygen atom. Therefore, if the molecule has 3 (of 4) hydrogen sites with undefined occupation and one with no hydrogen atom allowed, then the 3 sites have occupation 2/3 (to represent 3x2/3=2 hydrogen atoms). Point multipoles at these sites are multiplied by that occupancy. So far we have described the handling of the point multipoles from the same bonding network as molecules of the cluster. In the case of the other network there is no need to adjust occupancy and check for the presence or absence of any hydrogen atoms which serve as placeholders for point multipoles. They are placed at atoms of all molecules from that network which are no further than 8Å from the cluster for which wave function is calculated. The multipoles in that network are multiplied by atom site occupation (0.5 for each hydrogen site in this case).

7. DESY structure of ice VI

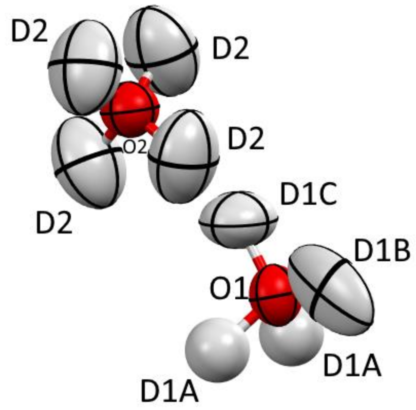

Figure 4S. Structure of D_2O was measured at DESY (Deutsches Elektronen-Synchrotron) at Petra III synchrotron facility, beamline P24. P24 is devoted to experiments on single crystalline samples under ambient or non-ambient conditions.

Table 4S. Comparison between neutron structure and X-ray structure for the synchrotron measurement at DESY. $\langle \Delta R \rangle$ - average difference in bond lengths between neutron and X-ray structure (Å), $\langle |\Delta U_{ij}| \rangle$ – Average absolute difference in components of ADPs, $\langle |\Delta U_{ij}|_{rel} \rangle$ – A relative average absolute difference in ADPs tensor components and $\langle S_{12} \rangle$ average similarity index. All ADPs data averaged over H-atoms only.

radiation	Neutron	X-ray DESY
Bond lengths (Å)		
O2 - D2	0.932(1)	0.87(4)
O1 - D1A	0.942(1)	0.91(3)
O1 - D1B	0.945(1)	0.79(6)
O1 - D1C	0.932(2)	0.94(4)
$\langle \Delta R \rangle$		0.06
$\langle |\Delta U_{ij}| \rangle$		0.0245
$\langle |\Delta U_{ij}|_{rel} \rangle$		0.85
$\langle S_{12} \rangle$		9.3

8. IAM structure of H_2O ice VI

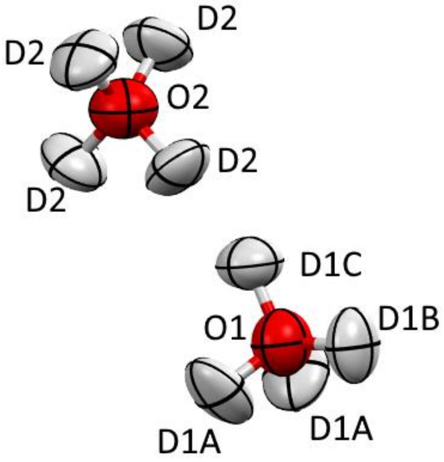

Figure 5S. Structure of H_2O was measured with an in-house diffractometer and refined with IAM model.

Table 5S. Comparison between neutron structure and X-ray structure for an in-house source. $\langle \Delta R \rangle$ - average difference in bond lengths between neutron and X-ray structure (Å), $\langle |\Delta U_{ij}| \rangle$ – an average absolute difference in components of ADPs, $\langle |\Delta U_{ij}|_{rel} \rangle$ – a relative average absolute difference in ADPs tensor components and $\langle S_{12} \rangle$ average similarity index. All ADPs data averaged over H-atoms only.

**radiation**	**Neutron**	**X-ray IAM**
Bond lengths (Å)		
O2 - H2	0.932(4)	0.737(15)
O1 - H1A	0.942(3)	0.760(11)
O1 - H1B	0.947(3)	0.755(16)
O1 - H1C	0.938(4)	0.736(18)
$\langle \Delta R \rangle$		0.192
$\langle \|\Delta U_{ij}\| \rangle$		0.078
$\langle \|\Delta U_{ij}\|_{rel} \rangle$		0.28
$\langle S_{12} \rangle$		3.03

9. APS structure of ice VI for HAR with model 1

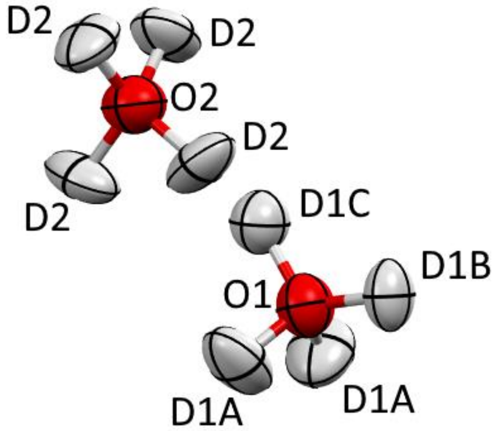

Figure 6S. Structure of D_2O was measured at APS (Advanced Photon Source) and refined with HAR using model 1 (described in the main text).

Table 6S. Comparison between neutron structure and X-ray structure for the synchrotron measurement at APS refined with HAR using model 1. $\langle \Delta R \rangle$ - average difference in bond lengths between neutron and X-ray structure (Å), $\langle |\Delta U_{ij}| \rangle$ – Average absolute difference in components of ADPs, $\langle |\Delta U_{ij}|_{rel} \rangle$ – A relative average absolute difference in ADPs tensor components and $\langle S_{12} \rangle$ average similarity index. All ADPs data averaged over H-atoms only.

**radiation**	**Neutron**	**X-ray HAR/model 1**
Bond lengths (Å)		
O2 - D2	0.932(1)	0.951(15)
O1 - D1A	0.942(1)	0.952(12)
O1 - D1B	0.945(1)	0.990(14)
O1 - D1C	0.932(2)	0.962(16)
$\langle \Delta R \rangle$		0.021
$\langle \|\Delta U_{ij}\| \rangle$		0.0065
$\langle \|\Delta U_{ij}\|_{rel} \rangle$		0.27
$\langle S_{12} \rangle$		2.59

10. References:

Gasser, T. M., Thoeny, A. V., Plaga, L. J., Koester, K. W., Etter, M., Boehmer, R. & Loerting, T. (2018). *Chem. Sci.* **9**, 4224–4234.

Komatsu, K., Noritake, F., Machida, S., Sano-Furukawa, A., Hattori, T., Yamane, R. & Kagi, H. (2016). *Sci Rep*. **6**, 28920.

Kuhs, W., Finney, J., Vettier, C. & Bliss, D. (1984). *J. Chem. Phys.* **81**, 3612–3623.

Militzer, B. & Wilson, H. F. (2010). *Phys. Rev. Lett.* **105**, 195701.

Rosu-Finsen, A., Amon, A., Armstrong, J., Fernandez-Alonso, F. & Salzmann, C. G. (2020). *J. Phys. Chem. Lett.* **11**, 1106–1111.

Rosu-Finsen, A. & Salzmann, C. G. (2019). *Chem. Sci.* **10**, 515–523.

Salzmann, C. G. (2019). *J. Chem. Phys.* **150**, 060901.

Salzmann, C. G., Radaelli, P. G., Mayer, E. & Finney, J. L. (2009). *Phys. Rev. Lett.* **103**, 105701.

Salzmann, C. G., Slater, B., Radaelli, P. G., Finney, J. L., Shephard, J. J., Rosillo-Lopez, M. & Hindley, J. (2016). *J. Chem. Phys.* **145**, 204501.

Shephard, J. J. & Salzmann, C. G. (2015). *Chem. Phys. Lett.* **637**, 63–66.

Thoeny, A. V., Gasser, T. M. & Loerting, T. (2019). *Phys. Chem. Chem. Phys.* **21**, 15452–15462.

Whale, T. F., Clark, S. J., Finney, J. L. & Salzmann, C. G. (2013). *J. Raman Spectrosc.* **44**, 290–298.

Whitten, A. E. & Spackman, M. A. (2006). *Acta Crystallogr. Sect. B-Struct. Sci.Cryst. Eng. Mat.* **62**, 875–888.
